# Integrating Microbiological Indicators and Shotgun Metagenomics for the Assessment of the Rhizosphere Microbiome of Medicinal Plants

**DOI:** 10.3390/ijms27135665

**Published:** 2026-06-23

**Authors:** Marta Wojtyś, Ewa Beata Górska, Ewa Osińska, Wojciech Stępień, Dariusz Gozdowski, Barbara Gworek, Angela Cunha, Isabel Natalia Sierra Garcia, Marek Kondras, Edyta Hewelke, Justyna Fidler-Jarkowska, Jarosław Chmielewski, Sławomir Orzechowski

**Affiliations:** 1 Division of Biophysics, Institute of Experimental Physics, Faculty of Physics, University of Warsaw, Pasteura 5, 02-093 Warsaw, Poland; mi.wojtys@uw.edu.pl; 2 Department of Biochemistry and Microbiology, Institute of Biology, Warsaw University of Life Sciences, Nowoursynowska 166, 02-787 Warsaw, Poland; ewa_gorska@sggw.edu.pl (E.B.G.); justyna_fidler@sggw.edu.pl (J.F.-J.); 3 Department of Vegetable and Medicinal Plants, Institute of Horticulture, Warsaw University of Life Sciences, Nowoursynowska 166, 02-787 Warsaw, Poland; ewa_osinska@sggw.edu.pl; 4 Stefan Batory Academy of Applied Sciences, Institute of Natural Sciences, Batorego 64C, 96-100 Skierniewice, Poland; wstepien@ansb.pl; 5 Department of Biometry, Institute of Agriculture, Warsaw University of Life Sciences, Nowoursynowska 166, 02-787 Warsaw, Poland; dariusz_gozdowski@sggw.edu.pl; 6 Department of Environmental Chemistry and Risk Assessment, Institute of Environmental Protection—National Research Institute, Słowicza 32, 02-170 Warsaw, Poland; barbara.gworek@ios.edu.pl; 7 Department of Biology, Center for Environmental and Marine Studies (CESAM), University of Aveiro, Campus de Santiago, 3810-193 Aveiro, Portugal; acunha@ua.pt (A.C.); inatalia.sierra@ua.pt (I.N.S.G.); 8 Department of Soil Sciences, Institute of Agriculture, Warsaw University of Life Sciences, Nowoursynowska 166, 02-787 Warsaw, Poland; marek_kondras@sggw.edu.pl; 9 Department of Environmental Development and Remote Sensing, Warsaw University of Life Sciences, Nowoursynowska 166, 02-787 Warsaw, Poland; edyta_hewelke@sggw.edu.pl; 10 Department of Public Health, Academy of Medical Sciences of Applied and Holistic Sciences in Warsaw, Al. Jerozolimskie 133A, 02-304 Warsaw, Poland; jaroslaw.chmielewski@amh.edu.pl

**Keywords:** rhizosphere microbiome, *Azotobacter*, allelopathy, *Allium ursinum*, plant–microbe interactions, soil microbiome

## Abstract

Medicinal plants are rich sources of bioactive secondary metabolites, yet their long-term effects on the rhizosphere (RS) microbial communities remain poorly understood, particularly with respect to microbial selection and functional potential. This study evaluated the number of selected groups of microorganisms culturable in vitro in the RS and bulk soil (BS) within 10-year monocultures of 11 medicinal plant species, and as a targeted case study, we performed shotgun metagenomic profiling for *Allium ursinum*. The abundance of microorganisms differed markedly among plant species, indicating species-specific RS selection. *Azotobacter* spp. showed the strongest variation: they were not detected in the RS of *Allium ursinum*, *Thymus vulgaris*, and *Carum carvi*, whereas higher counts were observed under *Artemisia dracunculus* (135.1 × 10^2^ CFU g^−1^ DM), *Melissa officinalis* (67.1 × 10^2^ CFU g^−1^ DM) and *Calendula officinalis* (38.8× 10^2^ CFU g^−1^ DM). *Azotobacter* spp. may serve as a sensitive candidate indicator of RS imbalance. Metagenomic analysis of the *A. ursinum*-associated soil revealed fine-scale taxonomic restructuring, while major functional categories remained broadly similar between the RS and BS. The novelty of this study lies in the development of the Integrated Microbiological Health Soil Index (IMHSI) and the proposal of a Nitrogen Enrichment Index (NEI) as exploratory composite metrics that integrate selected functional microbial groups.

## 1. Introduction

The global demand for medicinal and aromatic plants (MAPs) has increased in recent decades, driven by their essential applications in the pharmaceutical, cosmetic, and food industries [1,2,3]. As a result, the cultivation of these species has shifted from wild harvesting to controlled long-term monoculture systems. While the pharmacological properties of secondary metabolites produced by these plants—such as essential oils, flavonoids, and alkaloids—are well documented [4,5,6], their ecological impact on the belowground environment remains poorly understood. Medicinal plants function as active “chemical factories”, releasing significant quantities of bioactive compounds into the soil through root exudates, tissue decomposition, and volatilization [7,8]. Species such as *Salvia officinalis*, *Thymus vulgaris*, and *Allium ursinum* are increasingly used in biological systems because their metabolites may exhibit antimicrobial, insecticidal, fungicidal, allelopathic, and signaling activities [9,10]. However, in repeated monoculture systems, the continuous accumulation of these antimicrobial substances can exert strong selective pressure on the rhizosphere microbiome [11]. Previous ecological studies have shown that plant species identity and root exudation patterns are primary drivers of microbial community structure and function [12,13,14]. Mechanisms such as nutrient availability regulation [15,16] and carbon source allocation [17] play key roles in these interactions. Yet, the specific impact of bioactive metabolites from medicinal plants on rhizosphere microbial imbalance, allelopathic pressure, and microbiome-level responses remains a critical knowledge gap.

This concept is particularly relevant for long-term monocultures of medicinal plants, whose bioactive secondary metabolites may alter rhizosphere microbial equilibrium even in the absence of severe physicochemical degradation. Such deterioration typically proceeds as a cascade of interconnected processes. Repeated cultivation of the same plant species can lead to the accumulation of root-derived metabolites and crop residues, which, in turn, modify the rhizosphere chemical environment, including nutrient balance and pH. These changes act as ecological filters on the soil microbiome, promoting stress-tolerant or pathogenic taxa while reducing the abundance of beneficial microorganisms involved in nutrient transformation, stress adaptation, and plant-associated microbial homeostasis. As a result, long-term monoculture can weaken soil self-regulation, disrupt microbial interactions, and impair rhizosphere functioning. In medicinal plants, this cascade may be intensified by the continuous input of biologically active secondary metabolites with allelopathic and antimicrobial properties [18].

Despite increasing recognition of the soil microbiome as a cornerstone of sustainable agriculture and microbiome-mediated rhizosphere functioning [19], standard physicochemical analyses are often insufficient to detect early signs of biological degradation in the rhizosphere microbial communities associated with medicinal plants. Soil health is inherently linked to the functional capacity of the microbiome [20,21,22,23,24,25,26]. Therefore, there is a critical need to identify sensitive metagenomic and microbiological indicators that can diagnose the specific impact of medicinal plants on rhizosphere functionality before broad functional disruption becomes apparent. Recent advances suggest that integrating classical culture-dependent methods with modern metagenomic approaches provides a comprehensive assessment of rhizosphere microbial status [27]. While metagenomics offers insight into taxonomic shifts and potential functional stability [28], culture-dependent methods retain value by quantifying viable, physiologically active microbial guilds, such as aerobic free-living diazotrophs (*Azotobacter* spp.). *Azotobacter* spp. are particularly promising bioindicators due to their high sensitivity to environmental stress and chemical pollution [29], yet their response to herbal allelopathy remains underexplored.

In contrast to studies focused on single species, our work compares 11 noteworthy medicinal plant species, enabling us to identify species-specific patterns in the abundance of rhizosphere microorganisms. The novelty of this study lies in linking culture-dependent microbial indicators with shotgun metagenomic evidence to assess rhizosphere responses under long-term *Allium ursinum* monoculture, with particular emphasis on the transition from microbial stimulation to allelopathic suppression. A further element of novelty is the proposal of the Integrated Microbiological Health Soil Index (IMHSI) and the Nitrogen Enrichment Index (NEI) as exploratory composite metrics integrating selected functional microbial groups into a more synthetic interpretation of rhizosphere microbial status. In this context, the study offers an integrative framework for assessing metagenomic and microbiological indicators of rhizosphere responses in medicinal plant monocultures.

Hypothesis and Objectives. We hypothesized that the bioactive root exudates of medicinal plants exert species-specific selective pressure on the rhizosphere microbiome, inhibiting sensitive functional groups—specifically nitrogen-fixing bacteria—while maintaining overall metagenomic functional potential through taxonomic restructuring and broad functional similarity at the metagenomic level. To test this hypothesis, this study aimed to:

1. Evaluate rhizosphere responses under long-term monoculture of 11 distinct medicinal plant species by estimating the abundance of selected groups of microorganisms relevant to rhizosphere functioning using selective media.
2. Choose a sensitive microbiological index for the cultivation of medicinal plants.
3. Apply shotgun metagenomics to the rhizosphere of *Allium ursinum*—a species with strong antimicrobial properties—to assess whether microbial taxonomic shifts are accompanied by changes in metabolic potential.
4. Propose integrated microbial indices, specifically the Integrated Microbiological Health Soil Index (IMHSI) and the Nitrogen Enrichment Index (NEI), as exploratory tools for the integrated assessment of rhizosphere microbial status in medicinal plant monocultures.

## 2. Results

### 2.1. Modulation of Edaphic Properties by Long-Term Monoculture

The establishment of long-term monocultures led to significant heterogeneity in the soil matrix physicochemical profile (Table 1). While soil reaction (pH_KCl_) remained relatively stable across treatments (5.3–6.0), reflecting the high buffering capacity of the Eutric Cambisol, significant variation in nutrient stoichiometry was observed. Detailed analysis of the *Allium ursinum* niche (Table 2) identified the rhizosphere as a biogeochemical hotspot. Compared to bulk soil, the *Allium* spp. rhizosphere was significantly enriched in organic carbon (C_org_), potassium (+57%), and calcium (+45%). Notably, significantly elevated levels of sulfate sulfur (S-SO_4_) were retained in the rhizosphere (0.025% vs. 0.022% in bulk soil), directly correlating with the plant’s chemotaxonomic profile and the exudation of sulfur-containing secondary metabolites.

### 2.2. Host-Mediated Differentiation of Microbial Communities

Culture-dependent microbial communities differed significantly among the studied medicinal plant species (*p* ≤ 0.05), confirming that the rhizosphere effect varied in both magnitude and direction depending on the host plant (Table 3).

The abundance of heterotrophic bacteria (Lb) showed a particularly strong variation across rhizospheres. The highest value was recorded in the rhizosphere of *Allium ursinum* (3982.0 × 10^7^ CFU g^−1^ DW), while the lowest was observed for *Calendula officinalis* (16.0 × 10^7^ CFU g^−1^ DW). This contrast indicates substantial differences in the size of the culturable heterotrophic fraction among the studied plant species. In some cases, elevated bacterial counts in the rhizosphere may reflect copiotrophic enrichment and potentially greater availability of readily utilizable root-derived substrates [30]; however, this mechanism was not directly assessed in the present study.

The fungal component of the culturable microbiome also responded differently depending on plant species. The highest number of microscopic fungi (Lgm) was observed in the rhizospheres of *Allium ursinum* and *Carum carvi*, while lower values were found for the remaining plants. This indicates that plant-dependent rhizosphere effects were not limited to bacteria but also extended to fungal populations.

Distinct differences were also observed in the functional groups involved in organic matter decomposition. The highest abundance of cellulolytic microorganisms (LTMMC) was recorded in the rhizosphere of *Thymus vulgaris*, while amylolytic microorganisms (LTA) reached their highest values under *Allium ursinum*. These patterns suggest that medicinal plant species differ not only in their effects on total microbial abundance but also in the functional structure of the culturable microbiome.

### 2.3. Allelopathic Suppression of the Diazotrophic Guild

A critical ecological divergence was observed in the population of free-living nitrogen-fixing bacteria (*Azotobacter* spp.), distinguishing “Regenerative” from “Depletive” crops.

Stimulation via priming: Contrary to the hypothesis of a universal allelopathy, the rhizosphere of *Artemisia dracunculus* supported the highest density of *Azotobacter* spp. (Laz) (1351.0 × 10 MPN g^−1^ DM), significantly exceeding bulk soil populations (Table 3). Similar stimulatory trends were observed for *Melissa officinalis* and *Calendula officinalis*. This suggests that for these species, the metabolic benefit of carbohydrate-rich exudates outweighs potential chemical toxicity.

Intentional exclusion: In contrast, the elimination of *Azotobacter* spp. was observed in the rhizospheres of *Allium ursinum*, *Carum carvi*, *Thymus vulgaris*, *Saponaria officinalis*, *Arnica montana*, and *Salvia officinalis*. In these rhizospheres, the absence of *Azotobacter* spp. indicates a deliberate exclusion mechanism, likely due to the sensitivity of Gram-negative diazotrophs to various bioactive compounds, including antibiotics, saponins, and specific terpenes.

### 2.4. Multivariate Differentiation of Community Structure

**Figure 1:**
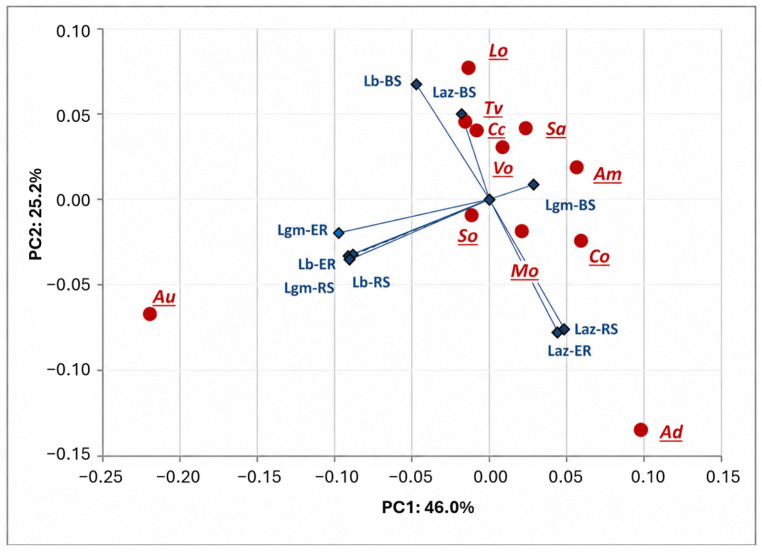
PCA results showing the multi-trait diversity of the studied objects and the relationship between the number of soil bacteria, microscopic fungi, and *Azotobacter* sp. in RS—rhizosphere and BS—bulk soil under the cultivation of the studied medicinal plants. *Legend:* Vo—Valerian (*Valeriana officinalis* L.), Au—wild garlic (*Allium ursinum* L.), Cc—caraway (*Carum carvi* L.), Lo—garden lovage (*Levisticum officinale* W.D.J. Koch), Tv—Thyme (*Thymus vulgaris* L.), Co—marigold (*Calendula officinalis* L.), Am—mountain arnica (*Arnica montana* L.), Mo—lemon balm (*Melissa officinalis* L.), So—soapwort (*Saponaria officinalis* L.), Sa—sage (*Salvia officinalis* L.), Ad—Tarragon (*Artemisia dracunculus* L.). Lb—number of bacteria; Lgm—number of microscopic fungi; Laz—number of *Azotobacter* sp.; BS—bulk soil; RS—rhizosphere; ER—rhizosphere effect.

**Figure 2:**
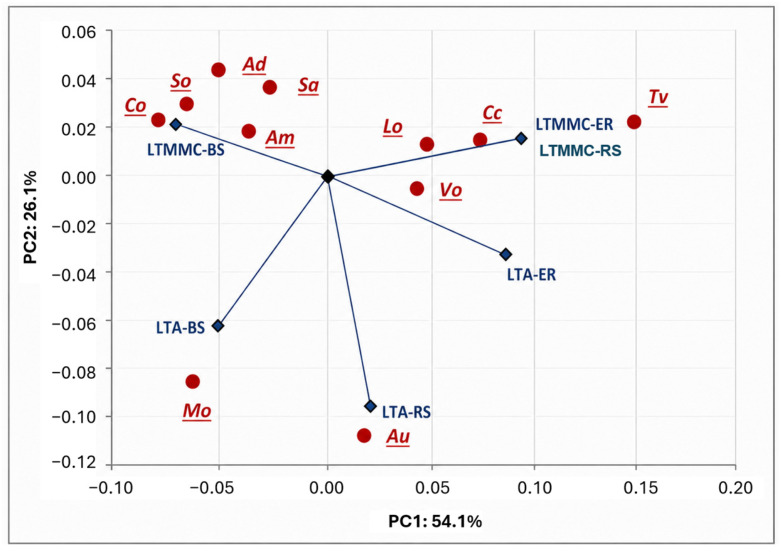
PCA results showing the multi-trait diversity of the studied objects and the relationship between the number of microorganisms involved in the decomposition of polysaccharides (cellulose and starch) in RS-rhizosphere and BS-bulk soil under the cultivation of the studied medicinal plants. Legend: Vo—Valerian (*Valeriana officinalis* L.), Au—wild garlic (*Allium ursinum* L.), Cc—caraway (*Carum carvi* L.), Lo—garden lovage (*Levisticum officinale* W.D.J. Koch), Tv—Thyme (*Thymus vulgaris* L.), Co—marigold (*Calendula officinalis* L.), Am—mountain arnica (*Arnica montana* L.), Mo—lemon balm (*Melissa officinalis* L.), So—soapwort (*Saponaria officinalis* L.), Sa—sage (*Salvia officinalis* L.), Ad—Tarragon (*Artemisia dracunculus* L.), LTMMC—number of aerobic cellulolytic microorganisms; LTA—number of aerobic amylolytic microorganisms; BS—bulk soil; RS—rhizosphere; ER—rhizosphere effect.

Principal Component Analysis (PCA) illustrated the multivariate differentiation of the studied objects. Figure 1 confirmed the formation of two distinct clusters: a “Diazotroph-Permissive Cluster” (dominated by *Artemisia dracunculus* L.) and a “Suppressive Cluster” (*Levisticum officinale*), corroborating the density data. Furthermore, PCA of polysaccharide decomposition (Figure 2) visualized a functional divergence driven by substrate quality. Plants with lignified root systems, such as *Thymus vulgaris*, were strongly correlated with cellulolytic microorganisms (LTMMC), whereas the fleshy, carbohydrate-rich bulbs of *Allium ursinum* favored amylolytic guilds (LTA).

### 2.5. Complementary Patterns Revealed by IMHSI and NEI

The synthetic indices provided a more integrated, yet still differentiated, view of rhizosphere microbial status across the studied rhizospheres (Table 4). The Integrated Microbiological Health Soil Activity Index (IMHSI) and the Nitrogen Enrichment Index (NEI) did not always change in parallel, indicating that they reflected partly different aspects of the microbial response.

In several cases, relatively high IMHSI values co-occurred with low or undetectable *Azotobacter* spp. counts (Table 4). This pattern was especially evident in the rhizosphere of *Allium ursinum*, where high heterotrophic abundance was accompanied by an absence of detectable *Azotobacter* spp. under the applied culture conditions (Table 3 and Table 4). In contrast, species such as *Artemisia dracunculus* and *Melissa officinalis* showed higher NEI values along with moderate or variably high IMHSI values (Table 4).

Overall, the results indicate that IMHSI and NEI captured different dimensions of rhizosphere microbial status, and their responses were not uniform across the studied rhizospheres.

### 2.6. Evaluation of Sequencing Depth and Species Richness

Shotgun sequencing yielded a total of 84,365,273 sequences, including 42,393,362 from bulk soil (BS) and 41,971,911 from the garlic rhizosphere (RS).

**Figure 3:**
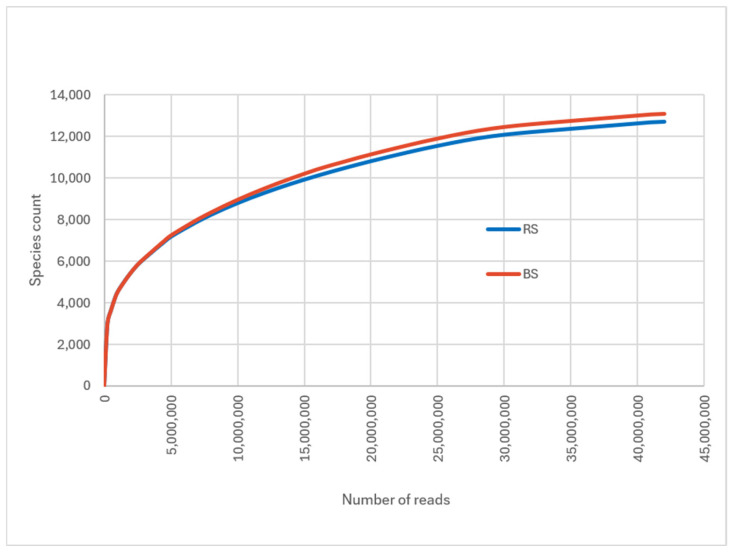
The rarefaction curves calculated at species count for RS—rhizosphere of garlic plants (blue color) and the bulk soil BS (red color).

To validate the coverage of the metagenomic dataset, rarefaction analysis was performed (Figure 3). The curves for both the *Allium ursinum* rhizosphere (RS) and bulk soil (BS) approached a saturation plateau at approximately 4.5 × 10^7^ reads, indicating that the sequencing depth was sufficient to capture the vast majority of the extractable microbial diversity, including rare taxa.

Notably, a clear separation in species richness was observed. The bulk soil (BS) red line) showed higher species richness (approximately 13,500 OTUs) than the rhizosphere (RS blue line, approximately 12,800 OTUs). This reduction in diversity within the rhizosphere supports the “Ecological Filtering Hypothesis”: although the root zone supports higher biomass, the chemical pressure of exudates selects for specifically adapted taxa, resulting in a slight decrease in overall alpha diversity compared to the more heterogeneous bulk soil.

### 2.7. Metagenomic Resolution of Rhizosphere Stability

**Figure 4:**
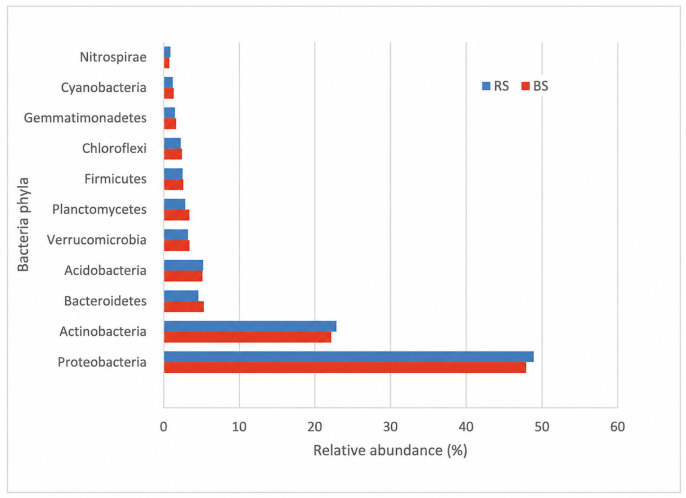
Relative abundance of dominant bacteria at phylum rank in bulk soil (BS) and rhizosphere (RS) soil of wild garlic.

**Figure 5:**
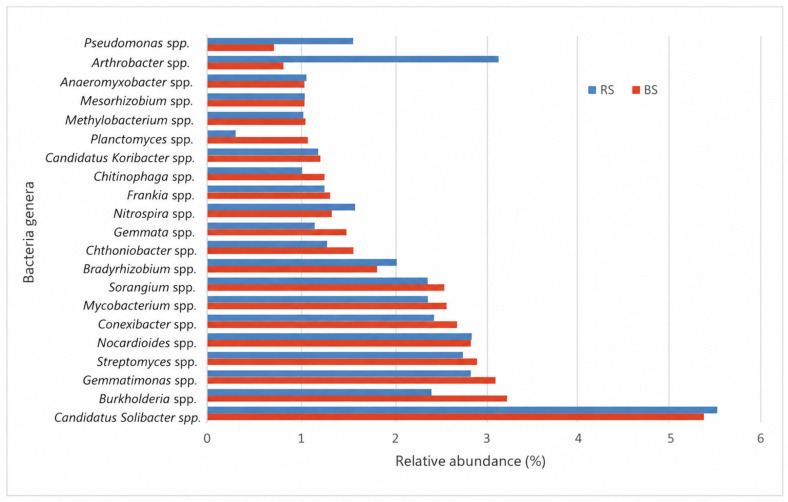
Relative abundance of dominant bacteria at the rank of genus in bulk soil (BS) and rhizosphere (RS) soil of wild garlic.

Metagenomic profiling of the *Allium ursinum* rhizosphere provided high-resolution insights into community structure, contrasting with culture-dependent observations. Unlike the fluctuations seen in culturable counts, the core microbiome showed remarkable stability at higher taxonomic ranks (Figure 4). The relative abundance of the dominant phyla, particularly Proteobacteria (~48%) and Actinobacteria (~22%), remained nearly identical between the rhizosphere (RS) and bulk soil (BS). This contradicts the hypothesis of broad-scale taxonomic dysbiosis. However, analysis at the genus level (Figure 5) revealed specific restructuring events within these broad groups. While the phylum-level structure remained intact, significant shifts occurred in the abundance of certain genera (e.g., the differential recruitment of *Arthrobacter* spp. and *Pseudomonas* spp.). This suggests that the rhizosphere effect in *Allium ursinum* monocultures operates through taxonomic fine-tuning, selecting for specifically adapted variants within major phyla rather than causing a community-wide collapse.

### 2.8. Functional Redundancy and Stress Adaptation

**Figure 6:**
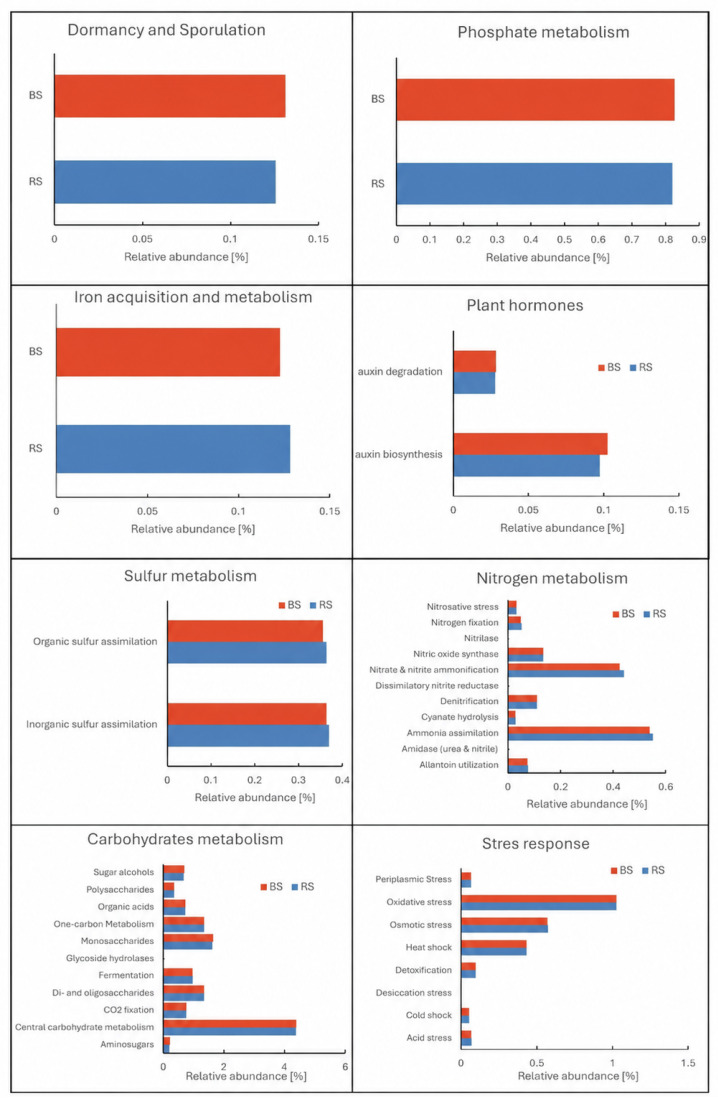
Relative abundance of sequences affiliated with functional SEED Subsystems levels in the rhizospheric (RS) and bulk soil (BS). Values of relative abundance refer to the total sum of genes found in each category in a metagenome, normalized by the number of total genes detected in that metagenome [dormancy and sporulation (SEED Subsytems Level 2), phosphate metabolism (SEED Subsytems Level 2), iron acquisition (SEED Subsytems Level 2), plant hormones (SEED Subsystems Level 3), nitrogen metabolism (SEED Subsystems Level 3), carbohydrate metabolism (SEED Subsystems Level 2), stress metabolism (SEED Subsystems Level 2), sulfur metabolism (SEED Subsystems Level 2)].

Functional annotation using SEED subsystems (Table 5, Figure 6) demonstrated a high degree of functional redundancy within the microbiome. Core metabolic pathways, including “Carbohydrates” and “Amino Acids and Derivatives,” showed no statistically significant difference in relative abundance between the rhizosphere and bulk soil (*p* > 0.05). This suggests that fundamental ecosystem services related to nutrient cycling are preserved.

However, a statistically significant upregulation (*p* < 0.05) was detected in the “Stress Response” (specifically oxidative stress defense) and “Sulfur Metabolism” subsystems. This indicates that while the community maintains metabolic homeostasis, it operates under chronic chemical stress imposed by the sulfur-rich organosulfur exudates of garlic [31,32]. The microbiome adapts by allocating increased genomic potential to detoxification and sulfur assimilation pathways, confirming a trade-off between growth and stress survival.

## 3. Discussion

### 3.1. Species-Dependent Rhizosphere Responses Under Medicinal Plant Monocultures

The present results show that medicinal plants may shape the rhizosphere microbiological community in a clearly species-dependent manner. Instead of producing a uniform response under long-term monoculture, the studied species were associated with distinct microbial patterns, suggesting that each plant creates a specific rhizosphere environment for microbial development (Table 3). This observation is consistent with the broader ecological view that plant identity and rhizosphere chemistry together structure soil microbial communities [12,13,14,22,23,33,34,35]. The total number of saprophytic bacteria in the rhizosphere of wild garlic (*Allium ursinum*) depends not only on the chemical compounds of root exudates but also on the physicochemical properties of the soil, as confirmed by the results of a study conducted by Borozan and Rab [36] on acidic forest soils in Romania. The researchers recorded only 120 CFU g^−1^ DM of saprophytic bacteria in the plant rhizosphere, which is drastically lower than our results from growing wild garlic in alluvial soil (Table 3). These findings directly support the usefulness of microbiological indicators for interpreting rhizosphere responses to medicinal plant monocultures.

A particularly interesting example was provided by *Allium ursinum*, which produces potent bactericidal substances (allicin and sulfur compounds) [37]; however, its rhizosphere showed high heterotrophic bacterial numbers, while low detectable counts of *Azotobacter* spp. were observed under the applied culture conditions (Table 3 and Table 4). The high abundance of microflora in the garlic rhizosphere suggests that bacteria have evolved resistance to these bactericidal substances and derive carbohydrate units (e.g., fructans) directly from the plant exudates as a carbon and energy source [38].

This contrast suggests that different microbial groups may respond differently to the same rhizosphere environment. One possible explanation is that some features of the rhizosphere may favor fast-growing heterotrophic microorganisms while being less suitable for more sensitive functional groups. However, because neither root exudate composition nor rhizosphere carbon fluxes were directly measured, this interpretation should be considered a plausible ecological explanation rather than a directly demonstrated mechanism.

More generally, the observed patterns indicate that microbial responses in medicinal plant monocultures are likely shaped by a combination of nutritional conditions, plant-derived bioactive compounds, and species-specific plant–microbe interactions. In this context, the results are better interpreted as evidence of selective rhizosphere structuring along a continuum from microbial stimulation to possible allelopathic suppression rather than as support for a single dominant process acting across all species. This broader interpretation is also consistent with the concept of soil fatigue as a biologically mediated deterioration of rhizosphere functioning under long-term monoculture [18].

### 3.2. Azotobacter spp. as a Sensitive Indicator of Rhizosphere Imbalance

The response of aerobic, free-living nitrogen-fixing *Azotobacter* spp. was among the most distinctive microbiological indicators observed in this study (Table 3 and Table 4). Compared with other microbial groups, *Azotobacter* spp. showed particularly strong variation among the studied rhizospheres, ranging from relatively high abundance in some plant species to undetectable counts in others under the applied culture conditions. This suggests that the diazotrophic component of the culturable microbiome may be especially sensitive to plant-dependent rhizosphere conditions.

A contrasting pattern was observed among the medicinal plant species. In the rhizospheres of *Artemisia dracunculus*, *Melissa officinalis*, and *Calendula officinalis*, *Azotobacter* spp. counts were relatively high, whereas in the rhizospheres of *Allium ursinum*, *Carum carvi*, *Thymus vulgaris*, *Saponaria officinalis*, *Arnica montana*, and *Salvia officinalis*, these bacteria were not detected (Table 3). Adamović et al. [39] determined the abundance of various microbial groups in the rhizosphere of medicinal and aromatic plants, including marigold (*Calendula officinalis*), and demonstrated a stimulating effect of the plant on the abundance of *Azotobacter* spp. in the marigold rhizosphere, which is also confirmed by the results of our investigation (Table 3).

These differences indicate that long-term medicinal plant monocultures may differentially affect diazotrophic microorganisms, possibly through a combination of rhizosphere chemistry, nutrient availability, and plant-derived bioactive compounds, as well as the multiplication of bacteriophages [40]. The ecological importance of *Azotobacter* spp. and their sensitivity to soil conditions have been emphasized previously, particularly in relation to nutrient status, pH, and environmental stress [29,41,42].

At the same time, the present data do not justify interpreting the absence of detectable Azotobacter as evidence of a complete functional collapse of nitrogen cycling. Rather, the results suggest that this group may serve as a particularly responsive microbial component reflecting biological imbalance in the rhizosphere. This interpretation is also supported by the partial divergence between *Azotobacter* spp.-based NEI values and the more general IMHSI scores (Table 4), indicating that the diazotrophic response and broader microbial abundance do not necessarily change in parallel.

### 3.3. Functional Differentiation of Decomposer Groups in the Rhizosphere

The studied medicinal plants also differed in their effects on microbial groups involved in the decomposition of polysaccharides (Table 3). This was especially evident for cellulolytic and amylolytic microorganisms, whose abundance varied substantially among rhizospheres. These differences suggest that medicinal plant species may influence not only total microbial abundance but also the functional structure of the culturable microbiome.

An interesting example was provided by *Thymus vulgaris*, whose rhizosphere supported relatively high counts of cellulolytic microorganisms, while the rhizospheres of *Allium ursinum* and *Melissa officinalis* were characterized by a high abundance of amylolytic microorganisms (Table 3). These differences may reflect plant-dependent variations in rhizosphere conditions and substrate availability. Specifically, the woody roots of thyme provide lignocellulose to the rhizosphere, while *Allium ursinum* provides starch, which induces the multiplication of cellulolytic and amylolytic microorganisms, respectively. However, the precise mechanisms behind these patterns could not be determined based on the current data. Similar ecological interpretations have been made in studies linking the abundance of specific microbial groups to organic matter turnover and the broader functional traits of the microbiome [21,43].

Taken together, these patterns indicate that medicinal plant species may create distinct microbial niches in the rhizosphere, reflected not only in total microbial abundance but also in the relative development of selected functional groups. However, because root morphology, substrate composition, and exudate chemistry were not directly measured in this study, these relationships should be interpreted as ecological associations rather than as directly demonstrated causal mechanisms.

### 3.4. Metagenomic Perspective on Rhizosphere Restructuring

The metagenomic analysis of soil under *Allium ursinum* provided an important complement to the culture-dependent microbiological indicators. While culture-based methods revealed pronounced differences in selected microbial groups (Table 3 and Table 4), the metagenomic data suggested a more stable overall functional picture. At higher taxonomic levels, the dominant phyla Proteobacteria (including classes: α-, β-, γ-, and δ-Proteobacteria), Actinobacteria, Bacteroidetes, and Acidobacteria remained broadly similar between the rhizosphere and bulk soil (Figure 4), whereas at finer taxonomic resolution, a restructuring of selected genera was observed (Figure 5). Yu et al. [44] demonstrated that rhizosphere soil under garlic (*Allium sativum* L.) cultivation in China was dominated by bacterial taxa from the phyla Proteobacteria, Chloroflexi, and Acidobacteria, findings that partially match our results. The dominant species in both studied soil zones were representatives of the genus *Candidatus solibacter*, which is consistent with the results of Yu et al. [44], followed by *Burkholderia* sp. and *Gemmatimonas* sp. (Figure 5). An increase in the relative occurrence of bacterial species of the genus *Candidatus Solibacter* and a decrease in *Burkholderia* sp. and *Pseudomonas* sp. were observed in the garlic rhizosphere. A positive effect on soil properties was also observed, namely an increase in the relative abundance of PGPR bacteria of the genera *Burkholderia* sp. and *Pseudomonas* sp. [45] in the *Allium ursinum* rhizosphere compared to the bulk soil.

Among the taxa at the rank of genus in the rhizosphere and in the bulk soil, the dominant representatives were the actinomycetes *Streptomyces* sp., *Nocardioides* sp., *Conexibacter* sp., *Mycobacterium* sp., *Frankia* sp., and *Arthrobacter* sp. Their relative occurrence in the rhizosphere, however, was lower, except for *Nocardioides* (Figure 5), which is an unfavorable phenomenon given the important functions that actinomycetes perform in agricultural ecosystems [46].

At the same time, the relative distribution of the main functional categories in the rhizosphere soil and the *Allium ursinum* bulk soil remained broadly comparable in both soil zones (Table 5; Figure 6).

This finding suggests that changes in selected culturable microbial groups do not necessarily translate into equally pronounced shifts in the overall metabolic profile of the soil microbiome. In other words, the rhizosphere of *Allium ursinum* appeared to undergo taxonomic restructuring while maintaining broad functional similarity at the metagenomic level. A similar distinction between taxonomic change and functional stability has been discussed in broader metagenomic and microbial ecology studies, which indicate that microbial communities may retain major ecosystem-related functions despite compositional shifts [21,28]. This supports the interpretation of the study as an integrated assessment of rhizosphere responses rather than as a conventional rhizosphere microbial status assessment alone.

For this reason, the metagenomic results are interpreted here as evidence of broad functional similarity despite taxonomic restructuring, rather than as proof of complete functional redundancy or resilience. This distinction is important because metagenomic potential does not necessarily correspond to actual gene expression or process rates in soil. The observed stability of major SEED functional categories (Table 5; Figure 6; Files S1 and S2) therefore complements, rather than contradicts, the culture-based evidence of selective shifts in sensitive microbial groups.

### 3.5. Implications for Rhizosphere Microbial Status Assessment in Medicinal Plant

The results of this study indicate that the biological assessment of the rhizosphere in medicinal plant monocultures, specifically regarding the microbial community and microbiome (*Allium* spp.) benefits from the combination of multiple lines of evidence focused on rhizosphere responses. Physicochemical parameters provide the necessary environmental context (Table 1 and Table 2), but they do not always capture microbiome-level differences among rhizospheres. In the present study, soils with relatively similar basic chemical characteristics differed markedly in the abundance of selected microbial groups (Table 1 and Table 3). This interpretation is consistent with the broader view that soil health and rhizosphere microbial status are closely linked to the taxonomic and functional organization of the microbiome and cannot be fully described by physicochemical properties alone [19,20,21].

In this context, the combined use of the IMHSI and the NEI appears informative because the two indices do not represent the same ecological dimension. The IMHSI integrates the abundance of selected microbial groups, thereby providing a more synthetic description of the culturable fraction of the rhizosphere microbiome. The NEI, in contrast, is based specifically on the response of free-living diazotrophs represented by *Azotobacter* spp., and thus reflects a narrower but functionally important component of the rhizosphere microbiome. The fact that the two indices did not always change in parallel (Table 4) suggests that an increase in the abundance of some microbial groups does not necessarily imply the maintenance of all ecologically relevant microbial functions. Similar caution in interpreting composite indices has been emphasized in studies on soil quality and rhizosphere microbiome assessment, where multiple biological variables are integrated but should still be read in relation to specific microbiome functions [47,48].

This point is particularly important for interpreting the rhizosphere of medicinal plants. In several cases, relatively high IMHSI values co-occurred with low or undetectable *Azotobacter* counts, indicating that a general increase in culturable microbial abundance may occur alongside the decline of a sensitive functional group. Conversely, rhizospheres with lower or moderate integrated microbial scores may still retain relatively high diazotrophic potential. Because *Azotobacter* is known to be sensitive to environmental stress and soil chemical conditions [29,41,42], their response may provide information not captured by general abundance-based indices alone. For this reason, the relationship between IMHSI and NEI should not be understood as a simple contrast between “beneficial stimulation” and “functional degradation,” but rather as a reflection of the fact that different microbial functions may respond differently to the same rhizosphere environment.

This interpretation also agrees with the broader ecological perspective that soil microbial communities can show uneven responses across taxonomic and functional levels [28,49]. In the present study, the divergence between culture-based indices (Table 4) and the relatively stable functional metagenomic profile of *Allium ursinum* soil (Table 5; Figure 6) suggests that function-specific microbial indicators may respond more sensitively than broad SEED-level functional categories. Accordingly, the IMHSI is best understood as an index of integrated culturable microbial potential, whereas the NEI reflects the response of a particularly sensitive diazotrophic group.

Importantly, the interpretation of these indices should remain proportional to the scope of the data. Neither IMHSI nor NEI should be regarded here as a fully validated diagnostic tool, and neither index alone is sufficient to define soil quality or rhizosphere functioning in absolute terms. However, when combined with culture-based counts, physicochemical context, and shotgun metagenomic profiling, they provide a useful framework for identifying species-specific differences in rhizosphere functioning under long-term medicinal plant monocultures. In this sense, the study supports the inclusion of metagenomic and microbiological indicators in the assessment of rhizosphere responses in medicinal plant rhizospheres, especially where plant-derived secondary metabolites may influence rhizosphere microbiome organization in subtle but ecologically relevant ways [18,19].

## 4. Materials and Methods

### 4.1. Study Location and Experimental Design

The field experiment was conducted at the experimental station of the Faculty of Horticulture, Warsaw University of Life Sciences (SGGW), Poland (52°09′38.6″ N 21°06′02.0″ E). The soil was classified according to the WRB system as an Eutric Gleyic Fluvic Cambisol (silt loam texture). Developed from Holocene alluvial deposits of the Vistula River, this soil is characterized by a high content of silt and clay fractions, which ensures favorable water retention capacity while maintaining adequate aeration. The study site has a temperate climate with an average annual temperature of 8.5 °C and an annual precipitation of 550 mm.

The experimental design included 11 species of medicinal plants cultivated in long-term monocultures (established 10 years before sampling) on 5 m^2^ plots, with three replications each: *Valeriana officinalis* L. (Valerian, Vo), *Allium ursinum* L. (Wild garlic, Au), *Carum carvi* L. (Caraway, Cc), *Levisticum officinale* W.D.J. Koch (Lovage, Lo), *Thymus vulgaris* L. (Thyme, Tv), *Calendula officinalis* L. (Pot marigold, Co), *Arnica montana* L. (Mountain arnica, Am), *Melissa officinalis* L. (Lemon balm, Mo), *Saponaria officinalis* L. (Soapwort, So), *Salvia officinalis* L. (Sage, Sa), *Artemisia dracunculus* L. (Tarragon, Ad).

### 4.2. Soil Sampling Strategy

Soil samples were collected during the active growing season (10 June 2015) from two distinct zones to assess the rhizosphere effect:

- Bulk Soil (BS): Composite samples were prepared from six random locations per plot, collected approximately 20 cm from the plant root system using an Egner stick (0–20 cm depth).
- Rhizosphere Soil (RS): Six plants per plot were excavated with their root balls. The root systems were shaken to remove loose soil, and the soil tightly adhering to the roots (approximately 1–2 mm) was recovered by washing with sterile distilled water.

Three representative samples of the rhizosphere, as well as the bulk soil taken from each plot, were prepared for chemical and microbiological analysis. For classical microbiological analyses, fresh samples were processed immediately. For metagenomic analysis, performed specifically for *Allium ursinum*, samples were flash-frozen on dry ice and stored at −80 °C until DNA extraction and were transported directly from the field to the Genomed S.A. Company in a refrigerated container for immediate processing.

### 4.3. Physicochemical Analysis

Selected physicochemical properties were determined in air-dried soil samples sieved through a 2 mm mesh. The analysis included:

- pH: Determined potentiometrically in 1 M KCl and H_2_O.
- Organic Carbon (Corg) and Total Nitrogen (N): Determined according to standard ISO protocols (PN-ISO 10,694 [50] and PN-ISO 11261 [51], respectively).
- Available Macroelements: Phosphorus (P), Potassium (K), Calcium (Ca^2+^), Magnesium (Mg), Sodium (Na), and Sulfur (S) were extracted with 0.03 M acetic acid according to the universal method described by Nowosielski [52], and the ion content was quantified using Inductively Coupled Plasma Atomic Emission Spectrometry (ICP-AES).

### 4.4. Culture-Dependent Microbiological Analyses

The abundance of specific physiological groups was determined using the serial dilution pour plate method on selective media.

- Soil heterotrophic bacteria (Lb): Grown on Bunt and Rovira’s medium [53] supplemented with the antifungal antibiotic cycloheximide (50 µg/mL).
- Microscopic fungi (Lgm): Grown on Martin’s medium [54] supplemented with streptomycin (50 µg/mL), which kills sensitive bacteria, and rose bengal (50 µg/mL), which inhibits the growth of streptomycin-resistant bacteria.
- Cellulolytic microorganisms (LTMMC): Cultivated on Dubos agar with 1% carboxymethylcellulose (CMC) [55]. Hydrolysis zones were visualized using 0.1% Congo red.
- Amylolytic bacteria (LTA): Grown on starch agar medium; hydrolysis was visualized using Lugol’s iodine [56]
- *Azotobacter* spp. (Laz): The Most Probable Number (MPN) of bacteria was determined using the three-tube method on Winogradsky’s nitrogen-free liquid medium. Additionally, to assess the presence of *Azotobacter* spp. in soil samples, both in bulk and in the rhizosphere, they were inoculated onto the surface of a Winogradsky’s nitrogen-free solid medium. After one week, results were evaluated based on macroscopic and microscopic observations of the cultures. The presence of *Azotobacter* spp. cells in the culture medium was confirmed on wet and negatively stained microscope slides. Bacterial cell counts were calculated using McCrady’s tables for triplicates [57].

All microbial cultures were incubated for 7 to 14 days (soil heterotrophic bacteria) at 28 °C.

### 4.5. Metagenomic Analysis

Total genomic DNA was extracted from the rhizosphere and bulk soil of *Allium ursinum* using the Genomic Mini AX Bacteria+ kit (A&A Biotechnology Innovating life Science, Gdańsk, Poland) with an additional mechanical lysis step (bead-beating), following protocols optimized for local soils [58]. DNA quality and the presence of bacterial DNA were verified by real-time PCR targeting the 16S rRNA gene with universal primers 1055F and 1392R [59].

Shotgun sequencing libraries were prepared using the NEBNext DNA Library Prep Master Mix (New England Biolabs, Ipswich, MA, USA) and sequenced on the Illumina HiSeq 4000 platform (San Diego, CA, USA). Raw reads were demultiplexed, quality-trimmed, and annotated using the MG-RAST server (Argonne National Laboratory (Lemont, IL, USA), University of Chicago (Chicago, IL, USA), and San Diego State University (San Diego, CA, USA)) with the SEED subsystems database [60].

### 4.6. Calculation of Soil Health Indices

To integrate the microbiological data into actionable rhizosphere microbial status metrics, the following synthetic indices were calculated:

#### 4.6.1. Integrated Microbiological Health Soil Index (IMHSI): Represents the Overall Microbial Potential

IMHSI = z(Lb) + z(Laz) + z(LTMMC) + z(LTA)

where z(x) is the normalized Z-score of the log-transformed abundance of each group.

To synthetically interpret rhizosphere microbial status, an index integrating the abundance of selected microbial groups was constructed. This index was calculated as the sum of standardized values (z-scores) of logarithmically transformed counts of soil heterotrophic bacteria (Lb), bacteria of the genus *Azotobacter* (Laz), cellulolytic microorganisms (LTMMC), and amylolytic microorganisms (LTA). This approach was adopted to integrate several microbiological parameters describing various aspects of soil microbiome functioning, such as total bacterial count, nitrogen transformation potential, and organic matter degradation capacity. Standardization of variables was necessary due to the different orders of magnitude and varying statistical variability of the analyzed parameters.

The approach is consistent with the commonly used methodology for constructing synthetic soil quality indices, in which individual biological parameters are first transformed and normalized, then aggregated into a single index describing the overall functioning of the soil ecosystem. A similar approach has been used in numerous studies assessing soil quality and microbiological activity [14,21,47,48,61,62,63].

#### 4.6.2. The Nitrogen Enrichment Index (NEI) Indicates the Potential for Non-Symbiotic Nitrogen Fixation [41]

NEI = log(Laz+ 1)

#### 4.6.3. Carbon Mineralization Index (CMI): Reflects the Efficiency of Organic Matter Decomposition [43]

CMI = LTMMC/Lb

#### 4.6.4. Fungal–Bacterial Index (FBI): An Indicator of Ecosystem Stability and Succession [64]

FBI = Lgm/Lb

### 4.7. Statistical Analysis

The significance of differences in microbial counts between plant species and soil zones (RS vs. BS) was assessed using one-way analysis of variance (ANOVA). Homogeneous groups were identified using Tukey’s HSD (honest significant difference) test at a significance level of α ≤ 0.05. Principal component analysis (PCA) was performed to visualize the multivariate relationships between microbial groups and plant species.

## 5. Conclusions

This study showed that long-term monocultures of medicinal plants were associated with clear, species-dependent differences in rhizosphere microbiome status, indicating that rhizosphere responses were not uniform. Among the analyzed microbial groups, *Azotobacter* spp. displayed particularly strong variation, and in several rhizospheres, its abundance remained undetectable under the applied culture conditions. This pattern suggests that *Azotobacter* spp. may be a sensitive candidate microbiological indicator of rhizosphere imbalance and possible allelopathic pressure in rhizosphere systems exposed to medicinal plant-derived bioactive inputs.

The integration of culture-dependent analyses with shotgun metagenomics provided a broader perspective on the organization and functional potential of the rhizosphere microbiome. In the case of *Allium ursinum*, marked shifts observed in selected culturable microbial groups co-occurred with broadly similar functional profiles at the metagenomic level. Because metagenomic profiling was performed only for *Allium ursinum*, these results should be interpreted as a species-specific case study that complements the culture-based analyses rather than as a direct representation of all medicinal plant species included in the experiment. This finding indicates that changes in sensitive microbial groups do not necessarily imply parallel changes in the overall metabolic potential of the rhizosphere microbiome.

For this reason, the Integrated Microbiological Health Soil Index (IMHSI) and the Nitrogen Enrichment Index (NEI) are interpreted here as exploratory complementary metrics reflecting different dimensions of rhizosphere microbial status rather than as fully validated diagnostic tools. Taken together, the results highlight the value of combining metagenomic and microbiological indicators to provide a more nuanced interpretation of rhizosphere responses at the microbiome level in medicinal plant monocultures, ranging from microbial stimulation to allelopathic suppression.

## Figures and Tables

**Table 1:** Selected chemical properties of the bulk soil (BS) on which the medicinal plants are grown, mean values (n = 3).

Plant	pH_KCl_	pH_H20_	C_org_%	N%	C:N
Vo	5.7 ab	5.89 a	1.39 a	0.146 a	9.52 ab
Au	5.3 a	5.54 a	1.38 a	0.141 a	9.79 ab
Lo	5.73 ab	5.99 a	1.44 a	0.159 a	9.06 a
Cc	6.02 b	6.19 a	1.39 a	0.152 a	9.14 a
Tv	6.00 b	6.16 a	1.42 a	0.139 a	10.22 b
Co	5.74 ab	5.86 a	1.47 a	0.157 a	9.36 a
Am	5.63 ab	5.82 a	1.32 a	0.124 a	10.65 b
Mo	5.84 ab	6.01 a	1.44 a	0.139 a	10.36 b
So	6.01 b	6.17 a	1.41 a	0.137 a	10.29 b
SzLSa	5.63 ab	5.92 a	1.35 a	0.127 a	10.63 b
Ad	5.79 ab	5.92 a	1.42 a	0.134 a	10.60 b

Vo—Valerian (*Valeriana officinalis* L.), Au—wild garlic (*Allium ursinum* L.), Cc—caraway (*Carum carvi* L.), Lo—garden lovage (*Levisticum officinale* W.D.J. Koch), Tv—Thyme (*Thymus vulgaris* L.), Co—marigold (*Calendula officinalis* L.), Am—mountain arnica (*Arnica montana* L.), Mo—lemon balm (*Melissa officinalis* L.), So—soapwort (*Saponaria officinalis* L.), Sa—sage (*Salvia officinalis* L.), Ad—Tarragon (*Artemisia dracunculus* L.), a, b—different letters next to the means indicate statistically significant differences in the columns at α ≤ 0.05.

**Table 2:** Selected chemical properties of tested soils under wild Garlic soil; mean values (n = 3).

Soil	pH	P	Ca^2+^	K	Mg	Na	C_org_	N	S	C:N Index	C:S Index
		mg/kg					%				
BS	5.3	88.1 a	1717.5 a	125.8 a	290.0 a	160.0 a	1.38 a	0.141 a	0.022 a	9.79 b	63.2 b
RS	5.5	89.7 a	2500.0 b	198.3 b	500.8 b	192.5 b	1.29 a	0.160 b	0.025 b	8.06 a	51.6 a

BS—bulk soil, RS—rhizospheric soil, P—phosphorus, Ca^2+^—calcium, K—potassium, Mg—magnesium, Na—sodium, C_org_%—organic carbon, N%—nitrogen, S%—sulfur, pH—acidity, different letters next to the means in column indicate significant differences at 0.05 significance level α ≤ 0.05.

**Table 3:** The number of microorganisms in 1 g of dry mass of soil under the cultivation of medicinal plants (n = 3).

Plant	(Lb)		(Lgm)		(Laz)		LTMMC		LTA	
	(RS) jtk × 10^7^	(BS) jtk × 10^5^	(RS) jtk × 10^3^	(BS) jtk × 10^2^	(RS) jtk × 10	(BS) jtk × 10	(RS) jtk × 10^7^	(BS) jtk × 10^4^	(RS) jtk × 10^6^	(BS) jtk × 10^5^
Vo	518.3 ab	58.7 abc	374.3 a	35.3 a	73.0 b	8.7 a	885.7 b	86.7 a	3585.3 ab	28.3 c
Au	3982.0 c	68.0 abc	10,278.0 b	17.7 a	0.0 a	5.4 a	47.3 a	78.7 a	13,487.3 c	26.3 bc
Lo	931.7 ab	89.3 c	18.3 a	43.3 a	62.0 b	32.0 c	674.0 b	99.0 ab	3139.7 a	10.1 ab
Cc	897.7 ab	72.0 bc	1174.0 b	44.7 a	0.0 a	18.0 b	646.3 b	89.0 a	2462.7 a	4.4 a
Tv	898.3 ab	92.3 c	550.0 a	40.3 a	0.0 a	2.5 a	1353.0 c	77.3 a	2934.3 a	4.6 a
Co	16.0 a	5.8 a	48.7 a	588.0 b	388.0 c	2.8 a	29.3 a	347.3 d	149.3 a	28.9 c
Am	58.3 ab	30.7 abc	25.7 a	2946.7 c	0.0 a	0.0 a	0.2 a	94.3 ab	18.3 a	21.3 abc
Mo	821.0 ab	53.3 abc	13.3 a	72.0 a	671.0 c	4.8 a	6.1 a	216.7 bc	8209.0 b	53.3 d
So	1051.3 b	30.7 abc	2.3 a	61.7 a	0.0 a	0.0 a	0.0 a	283.3 cd	3.0 a	21.7 abc
Sa	63.7 ab	82.0 c	23.3 a	96.0 a	0.0 a	0.0 a	0.5 a	89.0 a	4.2 a	8.9 ab
Ad	58.0 ab	9.0 ab	66.7 a	40.7 a	1351.0 d	1.1 a	2.9 a	303.3 cd	691.0 a	8.1 a

Vo—Valerian (*Valeriana officinalis* L.), Au—wild garlic (*Allium ursinum* L.), Cc—caraway (*Carum carvi* L.), Lo—garden lovage (*Levisticum officinale* W.D.J. Koch), Tv—Thyme (*Thymus vulgaris* L.), Co—marigold (*Calendula officinalis* L.), Am—mountain arnica (*Arnica montana* L.), Mo—lemon balm (*Melissa officinalis* L.), So—soapwort (*Saponaria officinalis* L.), Sa—sage (*Salvia officinalis* L.), Ad—Tarragon (*Artemisia dracunculus* L.), Lb—number of heterotrophic soil bacteria, Lgm—number of microscopic fungi, Laz—number of *Azotobacter* sp., LTMMC—number of aerobic mesophilic cellulolytic microorganisms, LTA—number of amylolytic microorganisms, RS—rhizosphere, BS—bulk soil, a, b, c, d—different letters next to the means indicate statistically significant differences in columns for α ≤ 0.05.

**Table 4:** Microbial indicators of soil biological activity under medicinal plant cultivation: Fungal-to-Bacteria Index (FBI), Nitrogen Enrichment Index (NEI), Carbon Mineralization Index (CMI), Integrated Microbiological Health Soil Index (IMHSI).

Plant	FBI		NEI		CMI		IMHSI	
	(RS)	(BS)	(RS)	(BS)	(RS)	(BS)	(RS)	(BS)
Vo	7.22 × 10^−5^	6.01 × 10^−4^	2.86	0.99	1.71	0.15	2.53	1.24
Au	2.58 × 10^−4^	2.60 × 10^−4^	0.00	0.81	0.01	1.16	2.13	0.91
Lo	1.96 × 10^−6^	4.85 × 10^−4^	2.79	1.52	0.72	1.11	2.76	1.27
Cc	1.31 × 10^−4^	6.21 × 10^−4^	0.00	1.28	0.72	0.12	1.05	−0.50
Tv	6.12 × 10^−5^	4.37 × 10^−4^	0.00	0.54	1.51	0.08	1.22	−1.38
Co	3.04 × 10^−4^	1.01 × 10^−1^	3.59	0.58	1.83	5.99	−0.81	0.52
Am	4.41 × 10^−5^	9.60 × 10^−2^	0.00	0.00	0.00	3.07	−3.58	−1.94
Mo	1.62 × 10^−6^	1.35 × 10^−3^	3.83	0.76	0.01	0.41	2.86	3.23
So	2.19 × 10^−7^	2.01 × 10^−3^	0.00	0.00	0.00	0.92	−4.72	−0.02
Sa	3.66 × 10^−5^	1.17 × 10^−3^	0.00	0.00	0.01	0.11	−3.89	−2.06
Ad	1.15 × 10^−4^	4.52 × 10^−3^	4.13	0.32	0.05	3.37	0.45	−1.29

Vo—Valerian (*Valeriana officinalis* L.), Au—wild garlic (*Allium ursinum* L.), Lo—garden lovage (*Levisticum officinale* W.D.J. Koch), Cc—caraway (*Carum carvi* L.), Tv—Thyme (*Thymus vulgaris* L.), Co—marigold (*Calendula officinalis* L.), Am—mountain arnica (*Arnica montana* L.), Mo—lemon balm (*Melissa officinalis* L.), So—soapwort (*Saponaria officinalis* L.), Sa—sage (*Salvia officinalis* L.), Ad—Tarragon (*Artemisia dracunculus* L.), RS—rhizosphere, BS—bulk soil.

**Table 5:** Functional composition of the soil metagenomes. Relative distribution (in percentage of annotated reads) of 28 major metabolic subsystems (using SEED subsystems in the MG-RAST program) detected in the rhizosphere (RS) and bulk soil (BS) metagenomes of wild garlic.

No	Metabolic Class	BS	RS
1	Carbohydrates	15.14	15.12
2	Clustering based subsystems	13.77	13.77
3	Amino Acids and Derivatives	11.44	11.45
4	Miscellaneous	7.24	7.25
5	Protein Metabolism	6.79	6.79
6	Cofactors, Vitamins, Prosthetic Groups, Pigments	6.45	6.45
7	RNA Metabolism	4.67	4.62
8	Fatty Acids, Lipids, and Isoprenoids	4.21	4.19
9	Cell Wall and Capsule	3.76	3.77
10	DNA Metabolism	3.71	3.66
11	Virulence, Disease and Defense	2.75	2.75
12	Respiration	2.71	2.74
13	Nucleosides and Nucleotides	2.47	2.46
14	Membrane Transport	2.26	2.27
15	Stress Response	2.24	2.26
16	Metabolism of Aromatic Compounds	1.93	1.97
17	Regulation and Cell signaling	1.20	1.21
18	Sulfur Metabolism	1.08	1.09
19	Phosphorus Metabolism	1.05	1.04
20	Cell Division and Cell Cycle	1.04	1.03
21	Phages, Prophages, Transposable elements, Plasmids	0.93	0.93
22	Nitrogen Metabolism	0.88	0.90
23	Motility and Chemotaxis	0.74	0.73
24	Iron acquisition and metabolism	0.60	0.61
25	Secondary Metabolism	0.39	0.39
26	Potassium metabolism	0.32	0.32
27	Dormancy and Sporulation	0.15	0.14
28	Photosynthesis	0.10	0.10

## Data Availability

The original contributions presented in this study are included in the article/Supplementary Materials. Further inquiries can be directed to the corresponding author.

## Supplementary Materials

The following supporting information can be downloaded at: https://www.mdpi.com/article/10.3390/ijms27135665/s1.

## Abbreviations

The following abbreviations are used in this manuscript:

Ad	*Artemisia dracunculus* L., tarragon
Am	Arnica montana L., mountain arnica
ANOVA	Analysis of variance
Au	*Allium ursinum* L., wild garlic
BS	Bulk soil
Cc	*Carum carvi* L., caraway
CFU	Colony-forming units
CMI	Carbon Mineralization Index
CMC	Carboxymethylcellulose
Corg	Soil organic carbon
DM	Dry matter
DNA	Deoxyribonucleic acid
DW	Dry weight
ER	Rhizosphere effect
FBI	Fungal-Bacterial Index
ICP-AES	Inductively coupled plasma atomic emission spectrometry
IMHSI	Integrated Microbiological Health Soil Index
Laz	Abundance of *Azotobacter* spp.
Lb	Soil heterotrophic bacteria
Lgm	Microscopic fungi
Lo	*Levisticum officinale* W.D.J. Koch, garden lovage
LTA	Number of amylolytic microorganisms
LTMMC	Number of aerobic mesophilic cellulolytic microorganisms
MAPs	Medicinal and Aromatic Plants
Mo	Melissa officinalis L., lemon balm
MPN	Most Probable Number
NEI	Nitrogen Enrichment Index
OTU	Operational Taxonomic Unit
PCA	Principal Component Analysis
PGPR	Plant growth-promoting rhizobacteria
RS	Rhizospheric soil
Sa	*Salvia officinalis* L., sage
So	*Saponaria officinalis* L., soapwort
Tv	*Thymus vulgaris* L., thyme
Vo	*Valeriana officinalis* L., valerian
WRB	World Reference Base for Soil Resources
